# Bone Marrow SSEA1+ Cells Support the Myocardium in Cardiac Pressure Overload

**DOI:** 10.1371/journal.pone.0068528

**Published:** 2013-07-09

**Authors:** Amanda Finan, Nikolai Sopko, Feng Dong, Ben Turturice, Matthew Kiedrowski, Marc S. Penn

**Affiliations:** 1 Summa Cardiovascular Institute, Summa Health System, Akron, Ohio, United States of America,; 2 Department of Integrative Medical Sciences, Northeast Ohio Medical University, Rootstown, Ohio, United States of America; Tokai University, Japan

## Abstract

**Rationale:**

Stage specific embryonic antigen 1+ (SSEA1+) cells have been described as the most primitive mesenchymal progenitor cell in the bone marrow. Cardiac injury mobilizes SSEA1+ cells into the peripheral blood but their *in vivo* function has not been characterized.

**Objective:**

We generated animals with chimeric bone marrow to determine the fate and function of bone marrow SSEA1+ cells in response to acute cardiac pressure overload.

**Methods and Results:**

Lethally irradiated mice were transplanted with normal bone marrow where the wild-type SSEA1+ cells were replaced with green fluorescent protein (GFP) SSEA1+ cells. Cardiac injury was induced by trans-aortic constriction (TAC). We identified significant GFP+ cell engraftment into the myocardium after TAC. Bone marrow GFP+ SSEA1 derived cells acquired markers of endothelial lineage, but did not express markers of c-kit+ cardiac progenitor cells. The function of bone marrow SSEA1+ cells after TAC was determined by transplanting lethally irradiated mice with bone marrow depleted of SSEA1+ cells (SSEA1-BM). The cardiac function of SSEA1-BM mice declined at a greater rate after TAC compared to their complete bone marrow transplant counterparts and was associated with decreased bone marrow cell engraftment and greater vessel rarefication in the myocardium.

**Conclusions:**

These results provide evidence for the recruitment of endogenous bone marrow SSEA1+ cells to the myocardium after TAC. We demonstrate that, *in vivo*, bone marrow SSEA1+ cells have the differentiation potential to acquire endothelial lineage markers. We also show that bone marrow SSEA1+ deficiency is associated with a reduced compensatory capacity to cardiac pressure overload, suggesting their importance in cardiac homeostasis. These data demonstrate that bone marrow SSEA1+ cells are critical for sustaining vascular density and cardiac repair to pressure overload.

## Introduction

Recent evidence suggests a role for a systemic stem cell response in the cardiac repair process [1,2], including a local cardiac stem cell (CSC) response [3–5]. In particular, bone marrow cells can be recruited to the heart after myocardial infarction or pressure overload (PO) [6,7]. The bone marrow cells arriving into the myocardium have the potential to replenish the CSC pool and contribute to angiogenesis [8,9]. However, the exact bone marrow stem cell populations involved and their specific roles in cardiac repair remain to be defined.

Stage specific embryonic antigen 1 (SSEA1) has historically been used as a marker of pluripotent stem cells, including embryonic stem cells, and was first described on blastomeres of the 8 cell stage embryo [10]. Populations of SSEA1+ cells have recently been identified in the adult, including in the bone marrow, spleen, heart, and brain [2,11,12]. Bone marrow SSEA1+ cells have been described as the most primitive mesenchymal progenitor cell with the potential to differentiate into all three germ layers in vitro [13]. Cell culture expanded SSEA1+ cells injected into mice have the potential to become mesenchymal and endothelial cells, and to a small degree, cardiac myocytes [12]. SSEA1 expression has also been associated with a specific multipotent bone marrow stem cell population known as very small embryonic-like stem cells (VSEL). VSEL are also identified as sca-1, oct-4, and CXCR4 positive [14].

Previous reports have described that exogenously delivered SSEA1+ cells after a myocardial infarction can improve cardiac function [15,16]. We have begun to extend the findings of SSEA1+ cell myocardial support by examining the response of the endogenous SSEA1+ cells in pressure overload. We have recently detailed the kinetics of systemic and cardiac SSEA1+ cells after cardiac pressure overload. We identified a concurrent rise of cardiac SSEA1+ cells with a decrease of SSEA1+ cells from the bone marrow and spleen suggesting the recruitment of peripheral SSEA1+ cells to the myocardium. In support of this, SSEA+ VSEL mobilize into the peripheral blood following myocardial infarction in mice and humans [17,18]. In combination, these reports support the hypothesis that peripheral SSEA1+ cells are recruited to the heart in cardiac repair but the fate and function of endogenous bone marrow SSEA1+ cells is still unknown.

To begin to address the previously undetermined role of the SSEA1+ bone marrow stem cells support to the heart, we generated unique chimeric mouse models to define the importance and function specifically of bone marrow SSEA1+ cells in response to cardiac pressure overload. We examined the effects of cardiac pressure overload on bone marrow SSEA1+ cell mobilization and differentiation. We provide evidence of bone marrow SSEA1+ cells involvement in the peripheral support to the cardiac remodeling process, with particular relevance to angiogenesis.

## Methods

### Animals

The animal work in this study was approved by the Institutional Animal Care and Use Committee of the Cleveland Clinic and Northeast, Ohio Medical University. Eight-week-old adult male C57BL/6J mice (Jackson Laboratories, 000664) were used for all experiments unless otherwise noted. Male age matched green fluorescent protein (GFP, Jackson Laboratories, 006567) were used for bone marrow transplantation studies.

### Bone Marrow Transplantation

Donor C57BL/6J and GFP transgenic mice were sacrificed by an overdose of ketamine and xylazine cocktail injected intraperitoneally. The hind limbs were removed at the femoral/pelvic joint and cleaned of any muscle. The femur and tibia were cut at both ends and flushed with 7-8 mL of flush media (Minimum Essential Medium Alpha Medium with 2 g/L NaHCO_3_, 10% horse serum, 10% FBS, 1% L-glutamine, 1% penicillin/streptomycin) with a 30-gauge needle and 10 cc syringe. Red blood cells were lysed from the collected bone marrow cells with red blood cell lysis buffer (155 mmol/L NH_4_Cl, 12 mmol/L NaHCO_3_, 0.1 mmol/L EDTA). Cells were then resuspended at 10^7^ cells/ml in flow cytometry buffer (1 mmol/L EDTA, 25 mmol/L HEPES pH 7.0, 1% FBS in PBS). Bone marrow cells were incubated for 30 minutes at room temperature with a fluorescently conjugated antibody against SSEA1 (PE or APC, BD Biosciences). SSEA1 positive and negative populations were sorted and collected on a FACSAria II (Becton-Dickinson). Purity was verified after each collection. Chimeric bone marrow was made by sorting GFP+ SSEA1+ cells into SSEA1 depleted bone marrow at the same SSEA1+ cells: total bone marrow ratio from prior to depletion. Recipient mice were pretreated with acidified antibiotic water (1.1 g/L neomycin, 1 million units/L polymixin, pH = 2.5-3.0) one week prior to transplant and maintained on the water until after total bone marrow reconstitution was verified. On the day of transplant, mice received 850 rads of irradiation (two doses of 425 rads of irradiation separated by four hours). The mice received one million bone marrow cells by intrafemoral injection into both legs. Four weeks after transplant, complete blood count analysis (Drew Scientific) was performed on blood from bone marrow transplanted mice to test for hematologic reconstitution. We have previously described this to be an efficient bone marrow transplantation protocol, resulting in >90% of the total bone marrow to be donor derived [19,20].

### Transverse Aortic Constriction

Transverse aortic constriction was performed as previously described [6]. Animals were anesthetized with a cocktail of ketamine (75 mg/kg) and xyalzine (7.5 mg/kg) injected intraperitoneally. Mice were attached to a Ventilator (Kent Scientific, rate: 105/min; limit pressure: 25 cm H_2_O; 100% O_2_, 1L/min) throughout the surgery. Under a dissecting microscope, sternotomy was performed and the transverse aorta between the left common carotid artery and the right subclavian artery was isolated. A 7-0 silk suture was constricted around the transverse aorta and an externally positioned blunted 27-gauge needle (~75% occlusion), which was removed after tightening. After banding, the sternum and skin were closed. Age-matched sham animals underwent the same procedure without tightening of the suture. Animals were administered buprenorphine (0.005–0.1 mg/kg) subcutaneously directly after surgery and twice daily for the following three days. The pressure gradient induced by the banding was evaluated one day after surgery by post wave Doppler echocardiography to ensure consistent banding (≥ 64 mmHg) between animals. Animals were sacrificed at 7 and 28 days after TAC was induced (n= 5-9 per group).

### Echocardiographic Assessment of Heart Function

Transthoracic echocardiography was performed using a Vivid 7 ultrasound machine (GE Medical) at baseline and 1, 2, and 4 weeks post-surgery. M-mode images of the hearts were captured. Ejection fraction, fractional shortening, diastolic and systolic left ventricular (LV) wall thickness, LV end diastolic dimensions (LVEDD), and LV end systolic dimensions (LVEDS) were measured. Echocardiographic measurements were performed and analyzed by investigators who were blind to the treatment allocation.

### Isolation of Heart Cells

Cardiac cells were isolated as described previously [8]. Briefly, heart tissue was minced and digested with 0.1% Collagenase B (roche Diagnostics), 2.4 U/mL Dispase II (roche Diagnostics), 500 KU/mL DNase I (roche Diagnostics), and 2.5 mmol/L CaCl_2_ for 30 minutes at 37°C. The cell suspension was filtered and washed with Hanks’ Balanced Salt Solution (HBSS) containing 2% fetal bovine serum (FBS) and 10 mmol/L HEPES.

### Flow Cytometric Analysis

Cell suspensions (10^7^ cells/ml in flow cytometry buffer) were incubated with a CD16/32 antibody to reduce non-specific binding antibody binding. Cardiac cell suspensions were incubated with LIVE/DEAD fixable near-IR stain (Invitrogen) for 30 minutes at room temperature. Cells were incubated with a biotinylated lineage enrichment cocktail (Stem Cell Techonologies) for 15 minutes at 4°C to identify stem cells from mature hematopoietic cell lineages. Cells were then incubated with fluorescently conjugated primary cell surface antibodies and streptavidin Pacific Blue (Invitrogen) for 30 minutes at room temperature. Combinations for stem cell populations were as follows: CSC – CD45, c-kit; HSC – c-kit, sca-1; EPC – flk-1, sca-1; SSEA-1 [9,21]. Fluorescently conjugated primary antibodies were purchased from BD Biosciences, excluding the SSEA-1 antibody that was purchased from R & D Systems. The appropriate conjugated isotype-matched IgGs were used as controls. Samples were run on a LSR II with FACSDiva software (BD Biosciences). The multi-color flow analysis was compensated with BD Compbeads (BD Biosciences) stained with the individual antibodies. Results were analyzed on FlowJo software (Version 9.3.3; Tree Star Inc).

### Histological analysis

Mice were euthanized with an overdose of xylazine/ketamine cocktail. The hearts were removed, flushed with saline, and weighed. The hearts were fixed for 24-48 hours in 10% neutral buffered formalin and then embedded into paraffin. Short axis tissue sections were cut at five microns. Collagen content was determined by Masson’s trichrome staining. Whole tissue sections were imaged by bright field microscopy (200x, Leica DM5000B). The percentage collagen positive area of the total section was measured to determine cardiac fibrosis (ImagePro 6.0).

### Immunohistochemistry

Paraffin sections were deparaffanized by xylene and rehydrated by sequential ethanol washes. Antigen retrieval was performed by heating of the slides for ten minutes in 10 nmol/L citrate buffer. Sections were incubated with fluorescein -conjugated isolectin (Vector Laboratories) to identify endothelium and Alexa Fluor 647 conjugated wheat germ agglutinin (Invitrogen) to label cardiac myocyte membranes. Five random fields were imaged at 400x for each section on the confocal microscope (Leica TCS SP5). Myocyte cell surface area was quantified on at least 60 myocytes per section (ImageJ). Vessel density (isolecin+/mm^2^) was quantified on five random fields per section (ImageJ). Samples were blinded and randomized prior to analysis.

Bone marrow cell and SSEA1+ cell derived cells engraftment in the myocardium was examined by immunostaining for GFP. Sections were incubated with fluorescein conjugated isolectin and a rabbit polyclonal antibody to GFP (Abcam, ab290). Alexa Fluor goat anti rabbit 568 secondary antibody (Invitrogen) was used to identify GFP. Sections were additionally stained with the endothelial lineage marker CD31 (Abcam, ab23864). Eight random fields per section were captured on a confocal microscope (630x) and GFP+ and Isolectin+ cells were evaluated.

### Statistics

Values are represented as mean ± SEM. Comparisons were evaluated using two-way ANOVA with Origin 7.0. *P* value <0.05 was considered statistically significant.

## Results

### Fate mapping of Bone Marrow SSEA1+ cells after TAC

**Figure 1:**
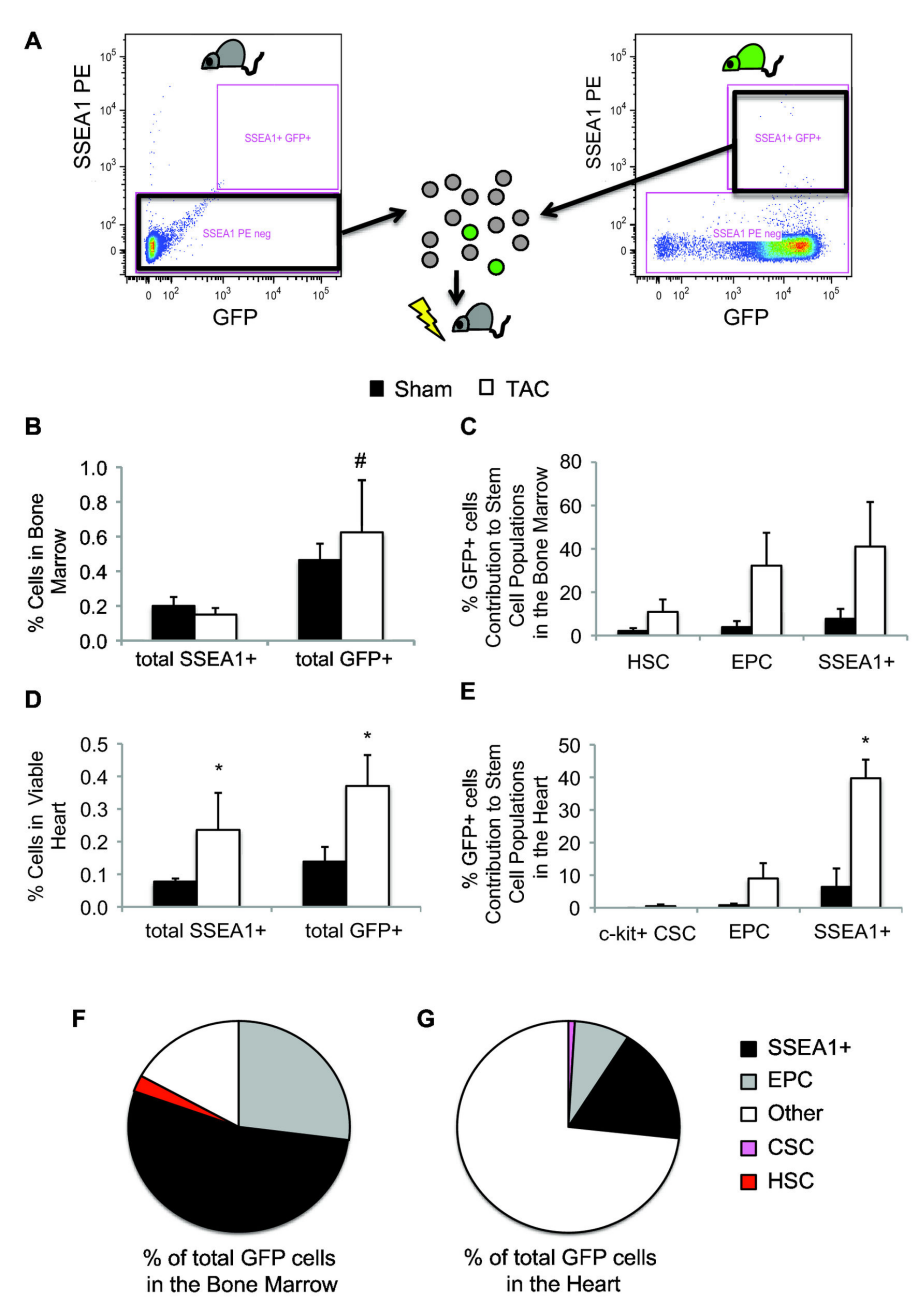
Fate of Bone Marrow SSEA1+ cells after TAC. (A) Experimental Protocol. SSEA1+ cells were depleted from the bone marrow (grey circles) of a C57 mouse (grey mouse) by FACS and replaced by bone marrow SSEA1+ cells from a GFP transgenic mouse (A1, green circles, green mouse). The chimeric bone marrow was injected into lethally irradiated C57 mice. Bar graphs from flow cytometry analysis of SSEA1+ and GFP+ cells in the bone marrow (B) and heart (D) from sham (black bars) and 7 days post-TAC (white bars). The percentage of GFP+ cells contributing to stem cell populations in the bone marrow (C) and heart (E) in sham and TAC (7 days post) animals was analyzed by flow cytometry. Bone marrow SSEA1+ cells contributed to the HSC and EPC, and SSEA1+ cell populations but not to the CSC population. The percentage of SSEA1+ (black wedge), CSC (pink wedge), EPC (grey wedge), and HSC (red wedge) of the total GFP+ cell pool in the bone marrow (F) and myocardium (G) 7 days post-TAC. n = 3-5 per group. * P < 0.05 compared to sham. # P = 0.05 compared to SSEA1+ cells. Abbreviations: SSEA1, stage-specific embryonic antigen 1; TAC, trans-aortic constriction; FACS, fluorescence-activated cell sorting; GFP, green fluorescent protein; HSC, hematopoietic stem cells; EPC, endothelial progenitor cells; CSC, cardiac stem cells.

We generated mice with chimeric bone marrow with the native SSEA1+ cells replaced by SSEA1+ cells from GFP transgenic mice (Figure 1A). Seven days after TAC or sham surgeries, the mice were sacrificed and their hearts and bone marrow were analyzed by flow cytometry. In the bone marrow, 7 days post-TAC, SSEA1+ cell levels were equal to sham animals (Figure 1B). However, significantly more GFP+ cells than SSEA1+ cells were found in the bone marrow of sham and TAC animals (SSEA1+ cells: 0.18 ± 0.03% of total BM, GFP+: 0.50 ±0.13% of total BM, P = 0.05). Flow cytometry analysis identified GFP+ cells in the hematopoietic stem cell (HSC) and endothelial progenitor cell (EPC) populations in the bone marrow of sham and TAC animals (Figure 1C).

We evaluated the recruitment of bone marrow SSEA1+ cells to the heart after TAC. We found an increase in the number of SSEA1+ cells 7 days post-TAC in the heart (Sham 0.08 ± 0.01% of viable cells, TAC 0.24 ± 0.11% of viable cells, P = 0.02, Figure 1D). The percentage of GFP+ cells in the myocardium was also elevated after TAC (Sham 0.14 ± 0.04% of viable cells, TAC 0.37 ±0.09% of viable cells, P = 0.04). We next examined whether various stem cell populations in the heart contained any bone marrow SSEA1+ derived GFP+ cells. The contribution of GFP+ cells to the c-kit+ CSC population was negligible (Sham 0.23 ± 0.23% GFP+/CSC+ of total CSC; TAC 0.40 ± 0.30% GFP+/CSC+ of total CSC; Figure 1E). A small proportion of GFP+ cells were identified in the EPC population, particularly after TAC. GFP+ bone marrow derived SSEA1+ cells were significantly elevated in the SSEA1+ cell population of the myocardium after TAC, and contributed to almost 40% of the total cardiac SSEA1+ cell population (39.71 ± 5.71% GFP+/SSEA1+ of total cardiac SSEA1+ cells, P = 0.01).

To further understand the fate of the bone marrow SSEA1+ derived GFP cells 7 days post-TAC, we defined the total GFP population by the various stem cell populations. In the bone marrow, more than half of the GFP+ cells (53.47% GFP+/SSEA1+ of total GFP, Figure 1F) were still SSEA1+ 7 days post TAC. Roughly a quarter of the GFP+ cells were positive for lineage markers of EPC (27%) while a small percentage were positive for HSC cell surface markers. As well, some bone marrow GFP+ cells were negative for any of the stem cell populations that we tested. In the heart, we identified that SSEA1+ cells (Figure 1G) and EPC accounted for a quarter of the total GFP+ population. The percentage of GFP+ cells that were positive for CSC markers was minor (0.94% of total GFP cells). The majority of the GFP+ cells (73.77%) in the heart 7 days post-TAC were negative for any of the stem cell populations that were tested.

### Cardiac function in response to TAC and bone marrow SSEA1 depletion

**Figure 2:**
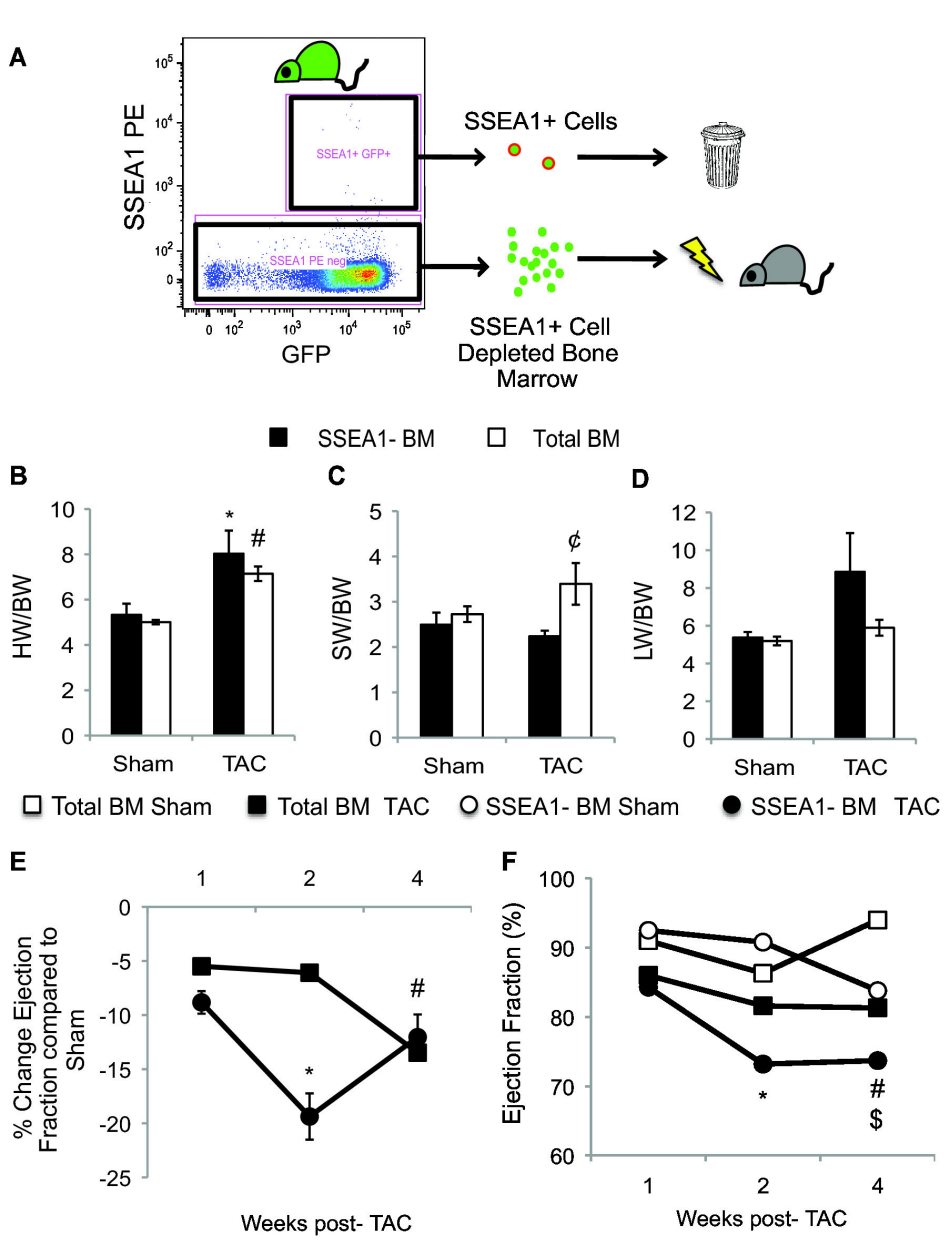
Bone Marrow SSEA1+ Cell Depletion Accelerates Cardiac Remodeling after TAC. (A) Experimental Protocol. SSEA1+ cells (green circles with red outline) were depleted from the total bone marrow of GFP transgenic mice by FACS and transplanted into lethally irradiated C57 mice (grey; SSEA1-BM). Control mice were transplanted with total GFP bone marrow (total BM). (B) Four weeks after TAC, the heart weight (HW/BW) was increased in both SSEA1-BM (black bars) and total BM (white bars) groups. (C) After TAC, the spleen weight (SW/BW) in SSEA1-BM mice was significantly lower compared to mice with total BM. (D) Measurement of lung weight (LW/BW) 4 weeks post-TAC. (E) The ejection fraction of SSEA1-BM mice (black circle) in response to TAC was decreased quicker relative to mice with total BM (black square). (F) The cardiac function of SSEA1-BM sham mice (open circle) decreased over time. Cardiac function of mice with total BM (open square) remained unchanged. n = 4-6. * P < 0.05 compared to SSEA1-BM sham. # P ≤ 0.01 compared to total BM Sham. ¢ P = 0.05 SSEA1-BM TAC versus total BM TAC. $ P = 0.04 SSEA1-BM Sham versus total BM Sham. Abbreviation: SSEA1, stage-specific embryonic antigen 1; TAC, trans-aortic constriction; GFP, green fluorescent protein; FACS, fluorescence-activated cell sorting; SSEA1-BM, SSEA1+ depleted bone marrow; BM, bone marrow; HW, heart weight; BW, body weight; SW, spleen weight; LW, lung weight.

We next transplanted lethally irradiated wild-type mice with GFP transgenic bone marrow depleted of SSEA1+ cells (SSEA-BM) to understand the importance of bone marrow SSEA1+ cells in cardiac repair (Figure 2A). Control mice were transplanted with complete GFP bone marrow (total BM). We allowed four weeks for full bone marrow reconstitution. No differences were measured in the complete blood counts of the transplanted animals regardless of the presence of SSEA1+ cells in the bone marrow (Supplemental Table 1).

TAC or sham surgeries were performed on transplanted mice and followed by echocardiography over a period of four weeks. Post-mortem analysis of the mice demonstrated that TAC significantly increased the heart weight to body weight ratio in both groups (SSEA1-BM 50.69 ± 11.11% increase over sham, total GFP BM 42.69 ± 2.73% increase over sham, P ≤ 0.05; Figure 2B). The spleen weight of the total BM group was elevated four weeks after TAC (Figure 2C). The spleen is a known storage site for HSC, SSEA1+ cells, and EPC and participates in the inflammatory process after acute myocardial infarction [1,9,22,23]. However, the spleen weight of the SSEA1-BM group was unchanged four weeks post-TAC and was significantly lower than the total BM counterparts (SW/BW 4 weeks post-TAC; SSEA1-BM 2.24 ± 0.12, total BM 3.39 ± 0.46, P = 0.05). Lung weight was also evaluated after TAC, but no significant changes were measured (Figure 2D).

The cardiac function of the SSEA1-BM mice decreased quicker after TAC compared to total BM mice. Ejection fraction (EF) was decreased by ~20 ± 2.14% two weeks post-TAC in SSEA-BM mice (Figure 2E, P = 0.04). In total BM mice, two weeks post-TAC, the EF was decreased by ~6 ± 0.70% compared to sham. However, four weeks after TAC, in both groups the EF was decreased by ~13% (P ≤ 0.01). At baseline, no significant differences in cardiac parameters were identified between the two groups; however as the experiment proceeded, the cardiac parameters of the sham SSEA1-BM mice began to decrease over time compared to the total BM sham mice (Supplemental Table 2). In particular, at four weeks after sham surgery, the ejection fraction of sham SSEA1-BM mice was significantly lower than its total BM counterpart (SSEA1-BM 83.75 ± 3.12%, total BM 94.00 ± 1.35%, P = 0.04; Figure 2F).

### SSEA1+ bone marrow depletion and vessel density after TAC

**Figure 3:**
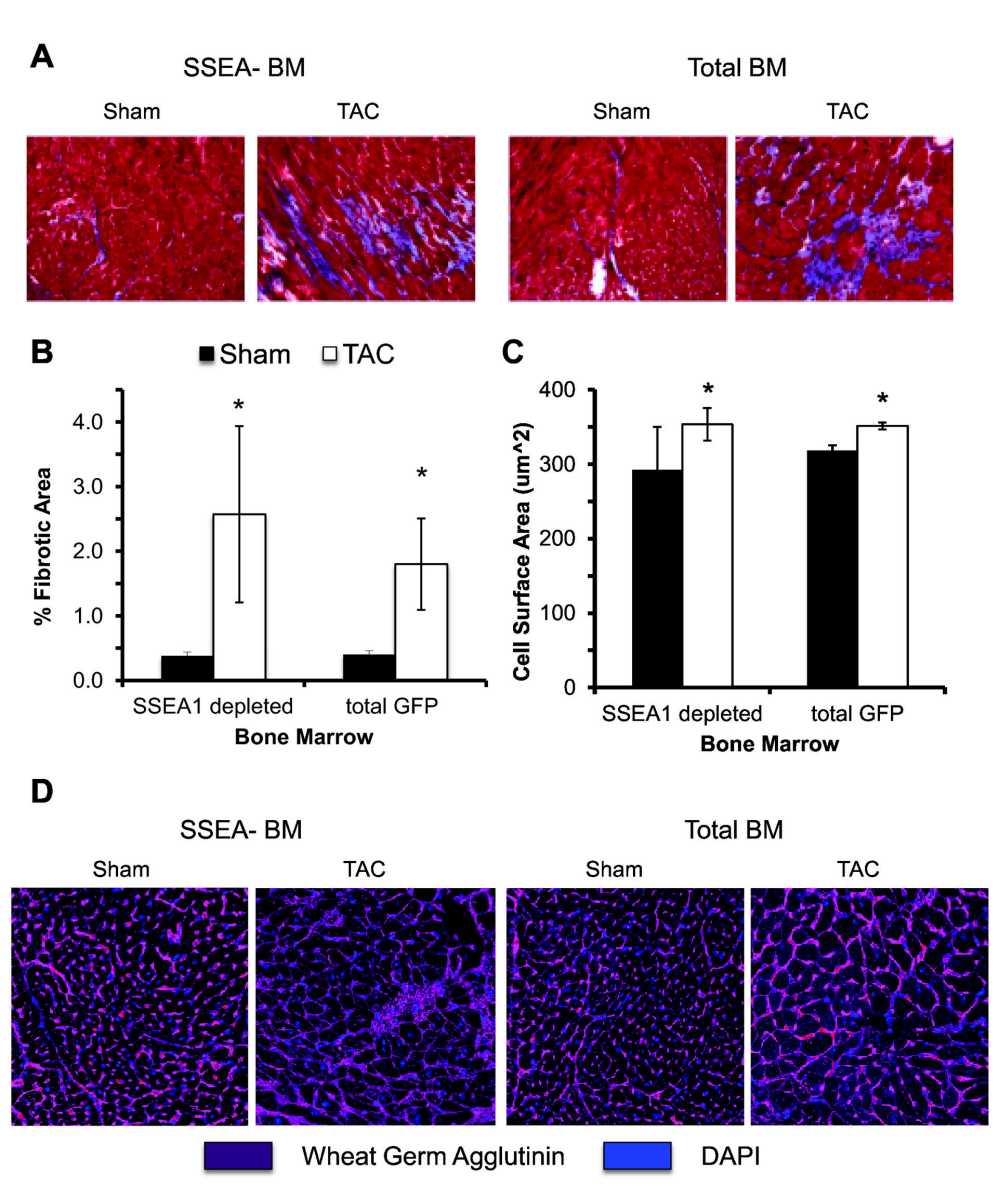
TAC increases fibrosis and **myocyte surface area.** (A) Mason’s trichrome staining for collagen (Magnification 20x). (B) TAC (white bars) significantly increased the presence of fibrosis in the hearts compared to sham (black bars) but no difference was observed based on bone marrow. (C) Four weeks after TAC, mice had increased myocyte hypertrophy compared to sham though no differences were determined between bone marrow. (D) Representative immunofluorescence of transversely sectioned myocytes of TAC and sham groups four weeks after surgery (40x Magnification). Myocyte membranes are outlined by WGA (purple) and nuclei are labeled with DAPI (blue). n = 4-6. * P ≤ 0.05 compared to sham. Abbreviations: TAC, trans-aortic constriction; WGA, wheat germ agglutinin; SSEA1, stage-specific embryonic antigen 1; SSEA1-BM. SSEA1+ cell depleted bone marrow; BM, bone marrow; GFP, green fluorescent protein.

We examined the effect of SSEA1 depletion on myocardial fibrosis and myocyte hypertrophy. TAC mice had significantly greater fibrosis compared to sham as determined by collagen content in the myocardium (P ≤ 0.05, Figure 3A & 3B). We identified an increase in cardiac myocyte surface area four weeks after TAC (P ≤ 0.03, Figure 3C & 3D). In both the measurement of fibrosis and cardiac myocyte hypertrophy, no differences were measured based on the presence of SSEA1+ cells in the bone marrow.

**Figure 4:**
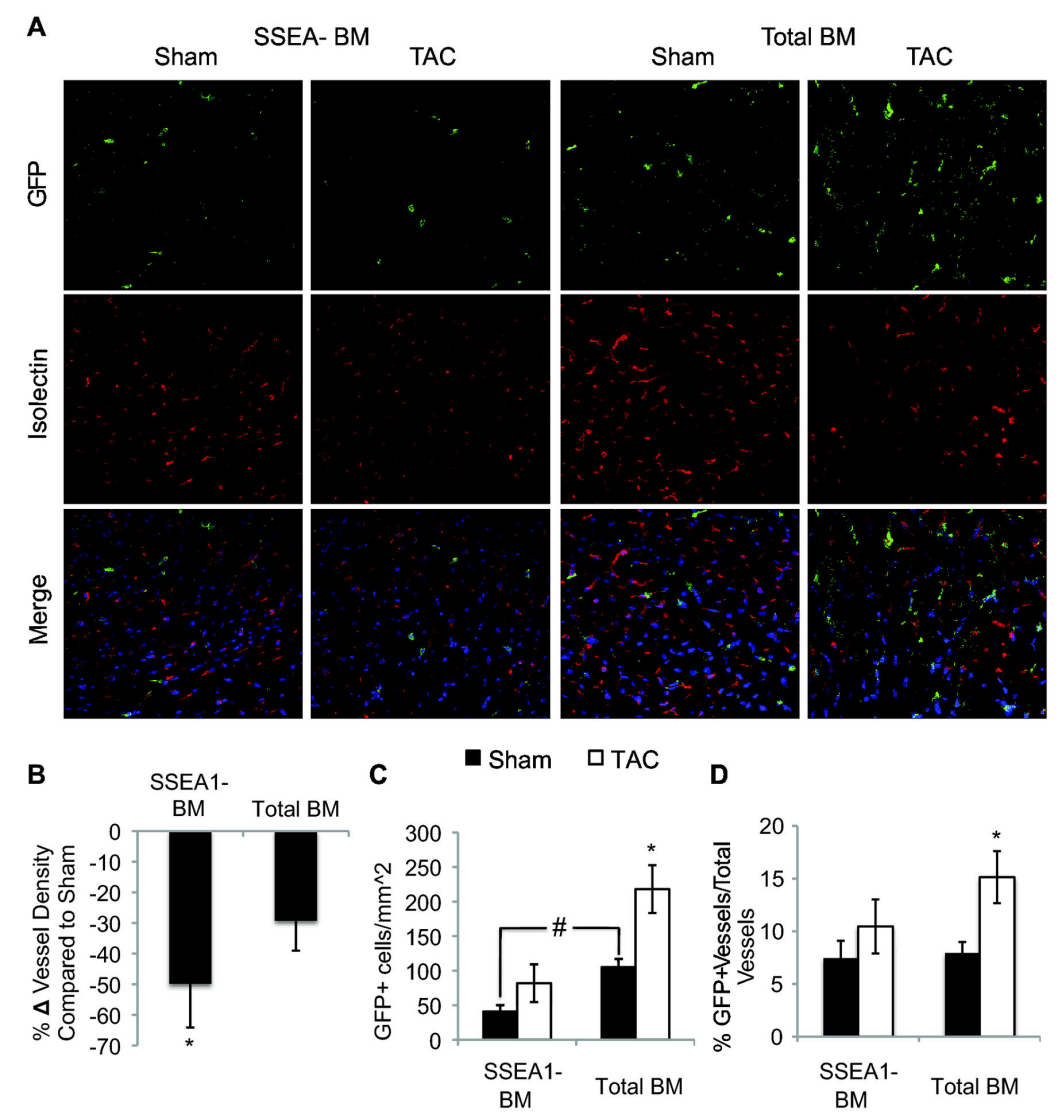
Depletion of SSEA1 from the Bone Marrow Increases Vessel Rarefaction after TAC. (A) Representative images used to quantify vessel density (Magnification 40x). Vessels were identified as Isolectin+ (Red) and nuclei are labeled with DAPI (blue). Representative images of GFP+ bone marrow derived (Green) alone and their co-localization with the vasculature (Isolectin, Red). Vessel density was quantified as Isolectin+/mm^2^. (B) Mice with SSEA1-BM had significantly decreased vessel density 28 days after TAC compared to sham (P = 0.03). No significant change was measured in TAC mice with total BM (P = 0.28). No differences were identified between sham animals. n = 4-6 per group. (C) Data representing the number of bone marrow derived cells (GFP+) in the myocardium in sham (black bars) and TAC (white bars) animals. In sham animals, there are significantly fewer bone marrow cells in the heart of SSEA1 depleted BM mice compared to animals with total BM. Four weeks after TAC, GFP+ cells were significantly increased in the myocardium in mice with total BM but not in SSEA1-BM mice. (D) Quantification of the percentage of total vessels that are GFP+ (bone marrow derived) in sham and TAC animals. The percentage significantly increased after TAC in mice with total BM compared to sham. No differences were measured between sham and TAC SSEA1 depleted BM mice or between sham groups. Abbreviations: SSEA1, stage-specific embryonic antigen 1; TAC, trans-aortic constriction; SSEA1 depleted BM, SSEA1+ cell depleted bone marrow; BM, bone marrow.

Based on our findings that SSEA1+ cells can acquire markers of EPC, we hypothesized that a decrease in vessel density in SSEA1-BM mice may account for their accelerated cardiac dysfunction. SSEA1-BM mice had increased vessel rarefaction four weeks after TAC compared to sham mice (-49.86 ± 14.26% compared to sham, P = 0.03, Figure 4B). Vessel density was decreased in TAC mice with total BM although it did not reach statistical significance (-29.30 ± 9.73% compared to sham). No significant differences in vessel density were found in sham animals.

### Total bone marrow cell recruitment to the myocardium after TAC in mice with SSEA1 depleted bone marrow

We next wanted to determine if the removal of SSEA1+ cells from the bone marrow would affect the recruitment of bone marrow cells to the myocardium after TAC. Localization of bone marrow cells (GFP+) in the myocardium of sham mice was significantly lower in SSEA1-BM mice compared to total BM mice (SSEA1-BM 47.85 ± 8.92 GFP+ cells/mm^2^, total BM 105.18 ± 11.92 GFP+ cells/mm^2^, P = 0.006, Figure 4C). Total BM mice had increased the number of bone marrow cells in the myocardium four weeks post-TAC compared to sham (107.24 ± 29.19% increase over sham, P = 0.028) (Figure 4C). Bone marrow cell recruitment to the myocardium after TAC in SSEA1-BM mice remained unchanged compared to their respective sham (Figure 4C).

Bone marrow cells can participate in cardiac angiogenesis [9,24,25]. We investigated the colocalization of bone marrow cells and vessels in the myocardium. An increase in the percentage of GFP+ (bone marrow derived) vessels in total BM mice after TAC was found (Sham 7.94 ± 1.05% *vs* 4 weeks post-TAC 15.14 ± 2.47%, P = 0.04, Figure 4D). After TAC, SSEA1-BM mice showed no change in bone marrow cell contribution to the vasculature compared to sham.

### Evaluation of bone marrow SSEA1 cells recruitment to the heart after TAC

**Figure 5:**
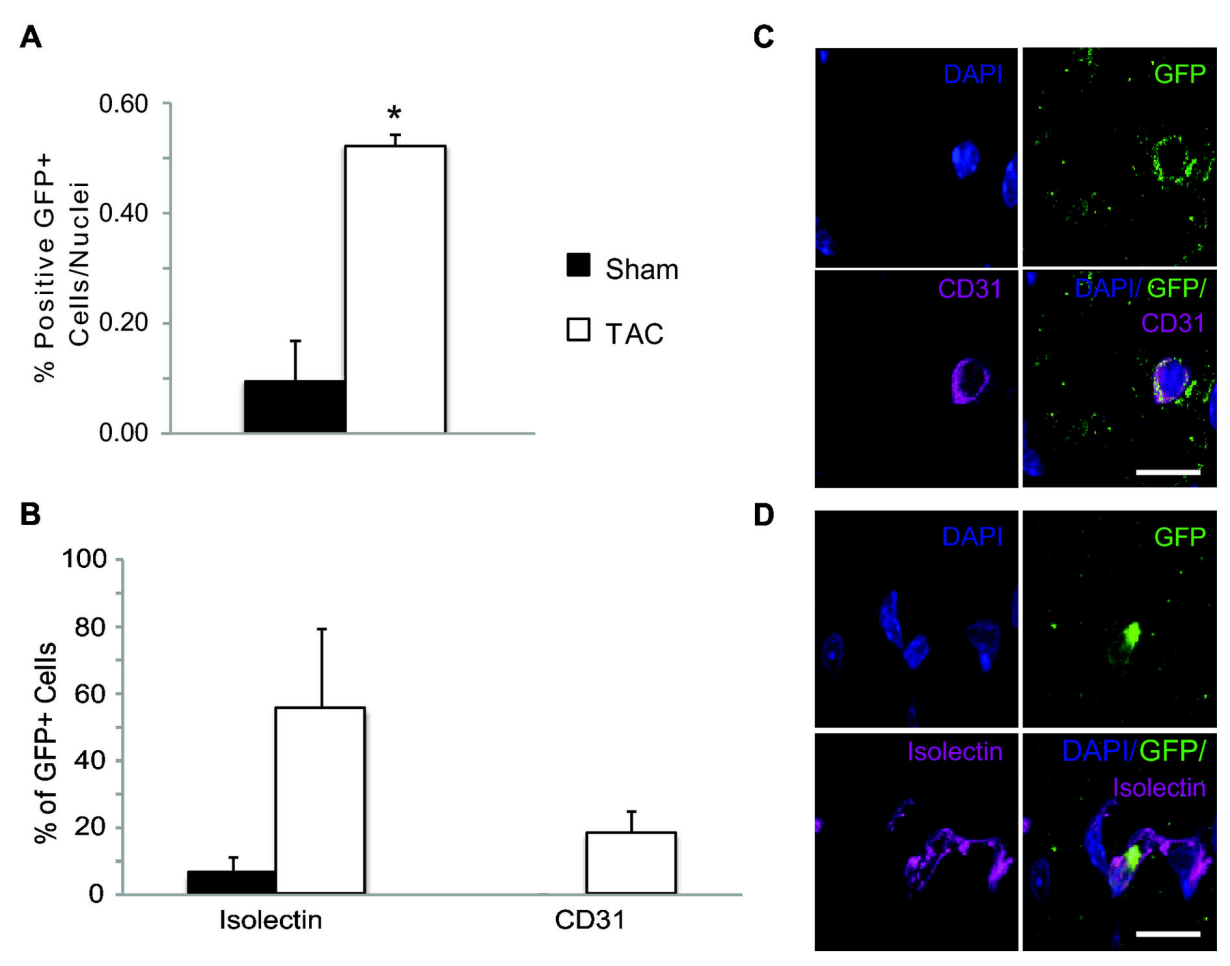
Bone Marrow Recruitment to the Heart is Reduced when SSEA1 Cells are Depleted from the Bone Marrow. (A) Bar graph representing the number of bone marrow cells (GFP+) in the myocardium in sham (black bars) and TAC (white bars) animals. In sham animals, there are significantly fewer bone marrow cells in the heart of SSEA1-BM mice compared to animals with total BM. Four weeks after TAC, GFP+ cells were significantly increased in the myocardium in mice with total BM but not in SSEA1-BM mice. (B) Quantification of the percentage of total vessels that are GFP+ (bone marrow derived) in sham and TAC animals. The percentage significantly increased after TAC in mice with total BM compared to sham. No differences were measured between sham and TAC SSEA1-BM mice or between sham groups. (C) Representative immunoflourescence images of bone marrow recruitment and bone marrow participation in angiogenesis in the myocardium (40x). Bone marrow cells are identified as GFP+ (red), vessels as Isolectin B4 + (green), and nuclei are labeled by DAPI (blue). n = 4-6. # P = 0.006 between sham animals. * P < 0.05 compared to respective sham. Abbreviations: SSEA1, stage-specific embryonic antigen 1; GFP, green fluorescent protein; TAC, trans-aortic constriction; SSEA1-BM, SSEA1+ cell depleted bone marrow; BM, bone marrow.

We repeated the generation of the chimeric bone marrow mice where only the SSEA1+ cells transplanted were GFP+. We induced TAC and sham surgeries on these animals and examined the long-term presence and fate of the bone marrow SSEA1+ cells in the myocardium four weeks after surgery. We identified a significant increase in the number of GFP+ cells in the heart four weeks after TAC (Sham 0.09 ± 0.07 GFP+ cells/nuclei vs 4 weeks post-TAC 0.52 ± 0.02 GFP+ cells/nuclei, P = 0.037, Figure 5A). We further determined that the bone marrow SSEA1+ cells acquired markers of endothelial lineage cells by identifying GFP+ cells colocalizing with CD31 and Isolectin B4 expression (Figure 5C and 5D). The percentage of GFP+ cells that expressed CD31 or Isolectin B4 was increased after TAC (Figure 5B).

## Discussion

Beyond the mobilization of SSEA1+ cells into the peripheral blood, little is known about the exact in vivo function of the endogenous SSEA1+ cell population [17,26]. Previous studies have demonstrated the myocardial support of exogenously delivered SSEA1+ cells [27]. In this study, we provide evidence for the involvement of endogenous bone marrow SSEA1+ cells in the pathophysiological changes occurring in the heart after the induction of cardiac pressure overload. We have used a unique approach to show evidence of endogenous bone marrow SSEA1+ cell mobilization to the pressure overload heart. We found that bone marrow SSEA1+ cells can adopt the cell surface markers of other stem cell populations and that TAC can have some affect on this. Further, we have clarified that bone marrow SSEA1+ cells do not contribute to the c-kit+ CSC population. We have also determined that the depletion of bone marrow SSEA1+ cells accelerates cardiac dysfunction after TAC, by mechanisms encompassing decreased bone marrow cell recruitment and increased vascular rarefaction. This study provides a clear demonstration for a critical role of the endogenous bone marrow SSEA1+ after cardiac pressure overload.

### Bone Marrow SSEA1+ cells can acquire markers of other stem cell populations

We identified a higher number of GFP+ cells compared to SSEA1+ cells in the bone marrow of sham and TAC animals. This suggested that SSEA1+ cells have the capacity to take on additional fates in a normal and pathological setting. We found a small portion of HSC were GFP+ (SSEA1 cell derived). Previous studies have described that VSEL (SSEA1+) can reconstitute the hematopoietic compartment when the cells were pre-expanded on stromal cells [28]. As we did not identify a large percentage of GFP+ cells in the bone marrow space, the GFP+ HSC may not be functionally participating in hematopoiesis. However, this result may be due to the early time point examined. It is also possible that although the GFP+ cells express markers of HSC, they may not be fully differentiated into HSC, potentially lacking the signaling required for the HSC to associate appropriately with its niche and receive the proper signaling cues [29,30]. In addition, we did not identify any significant changes in the complete blood cell counts in animals lacking bone marrow SSEA1+ cells. This suggests that even if SSEA1 derived HSC were identified, the GFP+ HSC were likely not an important participant in hematopoiesis.

We also identified bone marrow SSEA1+ derived GFP+ cells expressing the markers of EPC and endothelial cells. Further, depletion of SSEA1+ cells from the bone marrow decreased the vessel density in the myocardium after TAC. These evidences support bone marrow SSEA1+ cells having the capacity of acquiring an endothelial lineage. Our findings are corroborated by available evidence of transplanted SSEA1+ cells becoming endothelial cells [18,27,31]. The concurrent mobilization of peripheral SSEA1+ cells and EPC to the *loci* of cardiac injuries that we and others have described suggest SSEA1+ cells to be a source of EPC though functional significance of this event needs further experimental support [2,17].

The location of SSEA1+ cells in the hierarchy of bone marrow cells still needs to be determined. SSEA1+ cells have been previously described as multipotent cells and as the most primitive mesenchymal subset of cells [31,32]. The hemangioblast is a circulating multipotent progenitor cell in adults with the ability to give rise to hematopoietic and endothelial lineages [33]. Further studies will help determine if SSEA1+ cells are more primitive than hemangioblasts or if SSEA1+ cells are a different stem cell population with similar potency.

Similarly, we have identified bone marrow SSEA1+ cells to have overlapping characteristics with VSEL, including the mobilization after cardiac injury and the acquisition of endothelial and hematopoietic lineages [26,28,34]. It is possible that future experiments will identify that SSEA1+ cells and VSEL cells have additional commonalitites. If this will be found to be true, the isolation of a progenitor cell population by a simpler, more efficient method such as by a single marker (SSEA1) rather than a more involved flow cytometric protocol (VSEL) would lend itself to be more practical in a clinical situation.

In our study, we did not identify bone marrow SSEA1+ cells contributing to the c-kit+ CSC population. As well, we found very few c-kit+ CSC in the total cardiac GFP+ population. We focused on the c-kit+ definition of CSC as they are the only CSC population to provide all of the characteristics of a stem cell: self-renewal and multipotency [21]. It has been suggested that c-kit can also be a marker for cardiac fibroblasts; though it has recently been determined that purification of the CSC population with a c-kit antibody completely removes any contamination of fibrobasts in culture [35]. Regardless, we did not find bone marrow SSEA1+ cells acquiring c-kit expression suggesting that these cells do not differentiate into c-kit+ CSC or c-kit+ cardiac fibroblasts.

A significant number of bone marrow SSEA1+ cells were recruited to the myocardium after TAC enriching the myocardial SSEA1+ cell population. This data allows us to hypothesize that the bone marrow SSEA1+ cells may replenish the activated myocardial SSEA1 population. Previous reports have described the phenomenon of bone marrow cells contributing to the cardiac progenitor pool in the myocardium [7,36]. This is the first evidence that suggests a similar occurrence with SSEA1+ cells. Further studies will determine if bone marrow and resident myocardial SSEA1+ cells are phenotypically different and if these changes occur as the SSEA1+ cells are mobilized from the bone marrow.

We attempted to define the GFP+ cell population by various stem cell populations: SSEA1+, HSC, EPC, CSC. Of these stem cell groups, EPC and SSEA1+ cells accounted for the highest proportion of GFP+ cells. Interestingly, particularly in the heart, we identified GFP+ cells that were negative for the stem cell populations investigated. Based on the immunohistochemistry data, we can propose that a portion of the non-stem cell GFP+ cells are endothelial cells. It has been reported that transplanted SSEA1+ cells have the in vivo differentiation potential to become smooth muscle cells or other mesenchymal cells [12]. Endogenous bone marrow SSEA1+ cells may have a similar potential. There remains the possibility that the SSEA1+ cells acquire cell surface markers of other stem cell populations that were not investigated here, such as multipotent adult progenitor cells, mesenchymal stem cells, angioblasts, or other defined CSC populations such as sca-1+ CSC. EPC have been described to have a differentiation hierarchy though the surface markers have not been identified for the various phenotypes [37]. Due to the lack of the specific markers of differentiating EPC, we may have missed these cells in the transition from EPC to endothelial cells. Overall, this data further supports the notion that SSEA1+ cells have the ability to differentiate into other cell types. Future studies to define the non-stem cell GFP+ populations are already foreseen.

Splenomegaly occurs after TAC [2]. While this may in part be due to hemodynamic changes after TAC, we propose that the increase in spleen size may also be due to the proliferation and recruitment of stem cells; SSEA1+ cells and EPC in particular. Previous reports have placed the spleen as a central site for stem and inflammatory cell trafficking [1,9,38]. In this study, splenomegaly was not observed after TAC in animals that were depleted of bone marrow SSEA1+ cells. These current data support the hypothesis that the spleen may act as intermediate site for bone marrow derived stem cells to be recruited to sites of injury and places bone marrow SSEA1+ cells as participants.

### The myocardium is supported by bone marrow SSEA1+ cells

Our data demonstrate that bone marrow SSEA1+ cells play a direct, supportive role to the myocardium after TAC. Depletion of SSEA1 cells from the bone marrow resulted in an earlier decrease in cardiac function after TAC. We suggest this cardiac dysfunction to be a consequence of a decreased bone marrow cell recruitment and increased vessel rarefaction. Lower levels of bone marrow cells in the myocardium were also evident at 8 weeks post-bone marrow transplantation in sham SSEA1-BM mice. This pattern correlated to the decreased cardiac function in the SSEA1-BM sham animals compared to sham total BM animals. These data suggest that bone marrow SSEA1+ cells are also important for normal long-term cardiac function. SSEA1+ bone marrow cells may provide a steady state of cell renewal and maintain a baseline vascular density that is lost in the SSEA1-BM animals, resulting in cardiac dysfunction developing even in sham animals over a long period of time. If SSEA1+ cells are supplying endothelial lineage cells or additional cell types, the loss of these cells could account for the decreased function, as well as a smaller pool of bone marrow cells to support the myocardium. It is becoming increasingly more recognized that maximal cardiac repair requires not only a local response but systemic activation as well [39,40]. The benefits provided by the recruited bone marrow cells could include anti-apoptotic signals as well as pro-angiogenic signals [41]. Future experiments will examine if the transplant of SSEA1+ cells back into the animals can rescue the phenotype in SSEA1-BM mice.

We have recently described an early EPC and SSEA1+ cell response to TAC prior to any evidence of cardiac dysfunction but not a c-kit+ CSC activation [2]. Others have described elevated peripheral EPC levels, increased CSC in the myocardium, and decreased cardiac function in the late stages of cardiac pressure overload [9,42,43]. We now consider that endogenous stem cell activity could correspond to specific pathophysiological myocardial changes that occur in the setting of cardiac pressure overload; initially an increased metabolic requirement correlates with an angiogenic response (EPC and SSEA1+ cells), and later, as heart failure and dysfunction becomes evident and cardiac myocyte apoptosis begins, the CSC population gets activated [44]. This pathology differs from myocardial infarction where the injury is a quick and intense blow to the myocardium and billions of myocytes are lost. Endogenous stem cells are activated at a much quicker rate after an MI compared to TAC, particularly in the CSC population, and likely do not have the capacity to compensate for the extent of the damage [3,8,17]. In TAC, it is possible that the stem cells are able to compensate for a period but when this interval is over, the heart may begin to show signs of dysfunction. We propose that the phenotype of the SSEA1-BM TAC animals mimics the later period after TAC where cardiac compensation is lost. Studies such as this one begin to outline how the endogenous stem cells may participate in the transition from a compensated state to heart failure.

A large body of evidence supports the role of chemokine signaling in the recruitment of bone marrow cells in cardiac pathologies. Stromal derived factor-1 (SDF-1), monocyte chemotactic protein-3 (MCP-3), vascular endothelial growth factor, and fibroblast growth factor-2 have all been demonstrated to be elevated in the myocardium post-TAC [39,45,46]. SDF-1 and MCP-3, in particular, have been determined as strong bone marrow stem cell chemoattractant proteins in cardiac pathologies [47,48] and the over-expression of SDF-1 has recently been shown to improve clinical status in patients with chronic heart failure [49]. Interestingly, CXCR4 expression on SSEA1+ cells has been described as well as their migratory capacity when exposed to SDF-1 [50]. We currently hypothesize that the SDF-1/CXCR4 axis is an important signaling mechanism in the recruitment of bone marrow SSEA1+ cells to the myocardium after TAC but this needs to be validated in future experiments.

## Summary

Our results further our understanding of the *in vivo* fate and function of endogenous bone marrow SSEA1+ cells. Our data support that bone marrow SSEA1+ cells serve an important function for endogenous tissue repair and response to injury. We have further defined the in vivo differentiation potential for bone marrow SSEA1+ cells to transition to different fates, in particular to an endothelial lineage. Further understanding of the molecular mechanisms associated with bone marrow SSEA1+ mobilization, trafficking and differentiation may facilitate the development of new treatments for cardiac repair after injury.

## Supporting Information

Table S1 Complete blood counts following bone marrow engraftment in animals that received total or SSEA1 depleted bone marrow. (PDF)

Table S2 Echocardiographic parameters as a function of time after transaortic constriction (TAC) following bone marrow engraftment in animals that received total or SSEA1 depleted bone marrow. (PDF)
