# Supplementary material for: Bone Marrow SSEA1+ Cells Support the Myocardium in Cardiac Pressure Overload
Source: PLoS One. 2013 Jul 9;8(7):e68528. doi: 10.1371/journal.pone.0068528 (PMC3706399; doi:10.1371/journal.pone.0068528)
Supplement: Table S1 — (PDF) [file pone.0068528.s001.pdf]

Supplemental Table 1

	Total Bone Marrow (n = 8)	SSEA1 depleted Bone Marrow (n = 10)
White Blood Cells (K/ul)	3.61 ± 0.63	4.88 ± 0.98
Neutrophils (K/ul)	0.34 ± 0.04	0.49 ± 0.07
Lymphocytes (K/ul)	2.76 ± 0.52	3.24 ± 0.53
Monocytes (K/ul)	0.26 ± 0.09	0.21 ± 0.12
Eosinophils (K/ul)	0.28 ± 0.09	0.34 ± 0.09
Red Blood Cells (10^6/mL)	8.25 ± 0.38	8.44 ± 0.19
Hemoglobin (g/dL)	10.63 ± 0.50	11.5 ± 0.27
Hematocrit (%)	43.75 ± 1.80	47 ± 0.82
Platelets (K/ul)	703.75 ± 128.15	923 ± 54.92
