# Supplementary material for: Bone Marrow SSEA1+ Cells Support the Myocardium in Cardiac Pressure Overload
Source: PLoS One. 2013 Jul 9;8(7):e68528. doi: 10.1371/journal.pone.0068528 (PMC3706399; doi:10.1371/journal.pone.0068528)
Supplement: Table S2 — (PDF) [file pone.0068528.s002.pdf]

Supplemental Table 2

		IVS, d	IVS, s	LVID, d	LVID, s	LVPW, d	LVPW, s	Ejection Fraction
		(mm)	(mm)	(mm)	(mm)	(mm)	(mm)	%
1 week	TAC							
Total BM	-	1.15 ± 0.13	1.82 ± 0.07	2.56 ± 0.31	1.10 ± 0.14	1.13 ± 0.13	1.66 ± 0.04	91.00 ± 2.35
	+	1.21 ± 0.04	1.90 ± 0.18	2.56 ± 0.31	1.61 ± 0.21	1.07 ± 0.12	1.07 ± 0.12	86.00 ± 5.16
SSEA1-BM	-	1.07 ± 0.07	1.77 ± 0.04	2.87 ± 0.11	1.19 ± 0.11	1.07 ± 0.11	1.59 ± 0.10	92.50 ± 1.32
	+	1.11 ± 0.10	1.87 ± 0.18	2.97 ± 0.31	1.38 ± 0.44	1.02 ± 0.04	1.81 ± 0.13	84.33 ± 8.74
2 week								
Total BM	-	0.98 ± 0.05	1.65 ± 0.04	2.77 ± 0.08	1.39 ± 0.13	1.02 ± 0.10	1.42 ± 0.12	86.25 ± 3.57
	+	1.32 ± 0.04 *	1.86 ± 0.07 *	2.55 ± 0.19	1.37 ± 0.28	1.23 ± 0.07	1.46 ± 0.10	81.00 ± 6.00
SSEA1-BM	-	1.27 ± 0.06 †	1.87 ± 0.05 †	2.72 ± 0.13	1.21 ± 0.13	1.30 ± 0.08	1.68 ± 0.03	90.75 ± 1.89
	+	1.24 ± 0.12	1.74 ± 0.14	3.13 ± 0.31	1.99 ± 0.34	1.17 ± 0.09	1.58 ± 0.11	73.17 ± 6.56 *
4 week								
Total BM	-	1.20 ± 0.04	2.03 ± 0.10	2.61 ± 0.13	0.98 ± 0.10	1.01 ± 0.10	1.67 ± 0.05	94.00 ± 1.35
	+	1.25 ± 0.10	1.95 ± 0.15	2.77 ± 0.02	1.54 ± 0.08 *	1.25 ± 0.03	1.74 ± 0.07	81.33 ± 3.05 *
SSEA1-BM	-	1.15 ± 0.11	1.68 ± 0.16	2.64 ± 0.25	1.41 ± 0.17	1.20 ± 0.07	1.51 ± 0.10	83.75 ± 3.12 †

	+	1.13 ± 0.09	1.67 ± 0.22	3.02 ± 0.41	1.90 ± 0.53	1.07 ± 0.08	1.53 ± 0.10	73.67 ± 10.09
